# Lateral flow–based nucleic acid detection of SARS-CoV-2 using enzymatic incorporation of biotin-labeled dUTP for POCT use

**DOI:** 10.1007/s00216-022-03880-4

**Published:** 2022-01-19

**Authors:** Saloni Agarwal, Christian Warmt, Joerg Henkel, Livia Schrick, Andreas Nitsche, Frank F. Bier

**Affiliations:** 1 Institute for Biochemistry and Biology, Chair of Molecular Bioanalysis and Bioelectronics, University of Potsdam, Karl-Liebknecht-Strasse 24/25, 14476 Golm, Potsdam Germany; 2 Branch Bioanalysis and Bioprocesses, IZI-BB, Fraunhofer-Institute for Cell Therapies and Immunology, Am Mühlenberg 13, 14476 Golm, Potsdam Germany; 3 Centre for Biological Threats and Special Pathogens, Robert Koch Institute, Seestr. 10, 13353 Berlin, Germany; 4 Institute for Molecular Diagnostics and Bioanalysis-IMDB gGmbH, Veltener Str. 12, 16761 Hennigsdorf b, Berlin, Germany

**Keywords:** Point of care testing (POCT), Lateral flow assay (LFA), COVID-19, Reverse transcription loop-mediated isothermal amplification (RT-LAMP), SARS-CoV-2 N-gene

## Abstract

The degree of detrimental effects inflicted on mankind by the COVID-19 pandemic increased the need to develop ASSURED (Affordable, Sensitive, Specific, User-friendly, Rapid and Robust, Equipment-free, and Deliverable) POCT (point of care testing) to overcome the current and any future pandemics. Much effort in research and development is currently advancing the progress to overcome the diagnostic pressure built up by emerging new pathogens. LAMP (loop-mediated isothermal amplification) is a well-researched isothermal technique for specific nucleic acid amplification which can be combined with a highly sensitive immunochromatographic readout via lateral flow assays (LFA). Here we discuss LAMP-LFA robustness, sensitivity, and specificity for SARS-CoV-2 N-gene detection in cDNA and clinical swab-extracted RNA samples. The LFA readout is designed to produce highly specific results by incorporation of biotin and FITC labels to 11-dUTP and LF (loop forming forward) primer, respectively. The LAMP-LFA assay was established using cDNA for N-gene with an accuracy of 95.65%. To validate the study, 82 SARS-CoV-2-positive RNA samples were tested. Reverse transcriptase (RT)-LAMP-LFA was positive for the RNA samples with an accuracy of 81.66%; SARS-CoV-2 viral RNA was detected by RT-LAMP-LFA for as low as CT-33. Our method reduced the detection time to 15 min and indicates therefore that RT-LAMP in combination with LFA represents a promising nucleic acid biosensing POCT platform that combines with smartphone based semi-quantitative data analysis.

**Graphical abstract:**

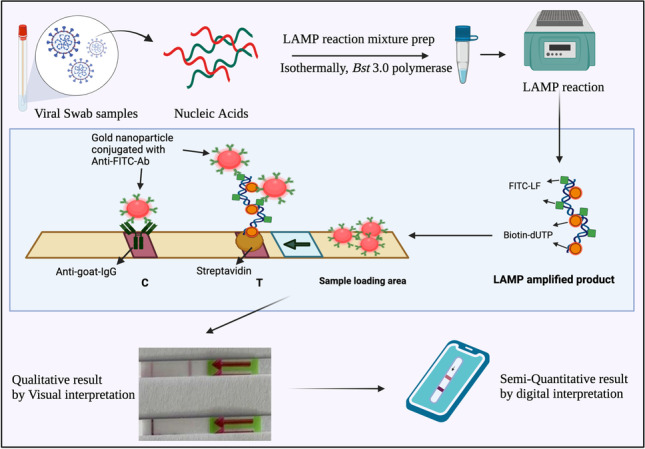

**Supplementary Information:**

The online version contains supplementary material available at 10.1007/s00216-022-03880-4.

## Introduction

The global COVID-19 pandemic has adversely affected humankind during the past 24 months since its first report in December 2019 in Wuhan, China [1, 2]. With the evolving mutant-variants and increase in their transmissibility, it is essential to design and develop ASSURED (Affordable, Sensitive, Specific, User-friendly, Rapid and Robust, Equipment-free, and Deliverable to end users) point of care diagnostics, as outlined by the World Health Organization (WHO) [3]. SARS-CoV-2 is an enveloped, positive single-stranded RNA virus, with ~ 30 kb of genetic material [4]. Mutations have been reported mainly in the genes encoding for the structural protein spike (S), envelope (E), membrane (M), ORF1ab, and nucleocapsid (N), which make the viral particle virulent and transmissible [5]. Out of all the variants or mutants of the virus, alpha (B.1.1.7), beta (B.1.351), gamma (P.1), delta (B.1.617.2), and omicron (B.1.1.529) variants are considered “variants of concern (VoC)” by the WHO due to their increased transmission rates and severity of infection.

When the antigen load is low in the sample, e.g., at an early stage of the infection, detection of the viral RNA is essential for infection diagnostics [6]. Hospitals and testing centers use RNA detection by RT-PCR as a gold standard diagnostic tool for SARS-CoV-2 detection in nasopharyngeal swabs [7]. RT-PCR is efficient in confirming the presence of viral nucleic acid in the samples, but it requires time, cost of equipment and material, and trained staff to perform the PCR tests [8, 9]. These limitations urge the development of more efficient point of care testing (POCT) for the diagnostics of SARS-CoV-2 [10]. Although rapid antigen-test kits are being widely used, their comparatively lower sensitivity still necessitates an increase in diagnostic efficacy by exploring new approaches of nucleic acid and antigen detection, resulting in more sensitive POCT [9].

Loop-mediated isothermal amplification (LAMP) has deliverable and reliable proof of concepts for a variety of DNA and RNA genomes from many microorganisms [11–13]. LAMP is an isothermal amplification technique, which may be advantageous over RT-PCR for nucleic acid amplification and detection [9]. LAMP provides robust, sensitive, and specific amplification of targets with less than 500-bp sequences. The novelty of LAMP and RT-LAMP is attributed to the polymerase, *Bst* 3.0, which acts as a “working horse” for the amplification [14]. The enzyme inherits three versatile and important properties: (a) strong strand displacement activity (no denaturation step required), (b) the lack of 5′–3′ exonuclease activity (concatemer, tandem multimer of target amplified DNA), and (c) enhanced reverse transcriptase activity (aiding in direct amplification of RNA) [15]. The enzyme’s strong strand displacement activity overcomes the necessity of temperature cycling of RT-PCR. Exploiting the reverse transcriptase property of the enzyme, RT-LAMP is also a suitable amplification method for RNA [16, 17]. Moreover, the RT-LAMP product may also be read out simply by using an immunochromatographic, paper-based lateral flow assays (LFA) when certain modifications are considered [18, 19]. However, previous LAMP research reports production of non-specific amplification products as a limitation affecting the specificity of results [9].

**Fig. 1:**
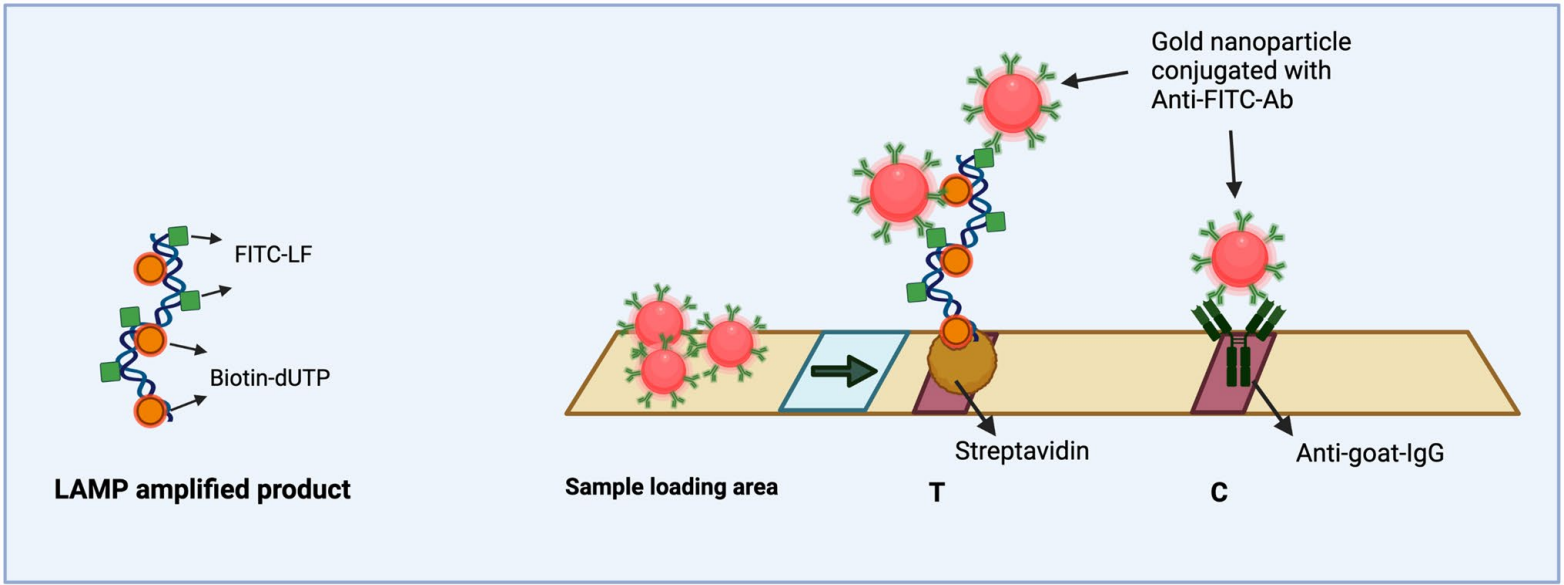
Schematic representation of the LAMP product visual readout via lateral flow assay (test strip from Milenia Biotech). Biotin and FITC molecules get incorporated into the product during amplification. The LAMP-amplified product is loaded on the sample loading area on the test strip. The anti-FITC-Ab–coated gold nanoparticles (GNP) bind with the LAMP product and the complex migrates up the test strip via capillary flow. The test line (T) appears on the strip only when the LAMP product is detected by GNP-Anti-FITC-Ab, whereas the control line (C) appears as an intrinsic control for the validity of the assay

LFA has been studied as a simple immunochromatographic readout platform for various target molecules and provides a versatile template for ASSURED POCT applications. For LFA readout, the target needs to be double-labeled, e.g., with FITC and biotin. Biotin is needed to attach the target to the surface of the LFA strip and FITC acts a capture moiety for anti-FITC-antibodies immobilized on gold nanoparticles (GNPs) present on the test strip. The agglomeration of GNPs on the test strip produces visible test and control lines (Fig. 1). In contrast, a recent report by Tan and coworkers [19] provides a proof of concept study with a test strip setup, where the nanoparticles for visualization are covered with streptavidin and the test line contains anti-FITC-antibodies; the control line uses biotin. While Tan et al. do not disclose the source of the LFA used in their study, we chose a standardized, commercially available LFA template, where the capture proteins (streptavidin for the test line and anti-IgG antibodies for the control) are already fixed.

In this study, we present a proof of concept for an RT-LAMP-LFA for RNA of SARS-CoV-2. LAMP was modified to enable a highly sensitive and specific amplification. We reduce the non-specificity of LFA readout by introducing certain modifications mentioned in “LAMP modifications.” We enhanced the polymerase activity by the incorporation of helicase and reverse transcriptase (RTase) as a consortium of enzymes. Helicase, a DNA unwinding enzyme, helped in reducing non-specific amplifications due to primer dimer background, whereas the RTase helped in enhancing the RTase activity of *Bst* 3.0 polymerase. This approach boosts the reaction speed, and processing time of a sample may be dropped to 15 min, while the single-enzyme system reported by Tan et al. needs at least 30 min. Using this modified technique, we confirm the detection of SARS-CoV-2 by using N-gene as the target and employing 3 specific primer sets F3-B3, FIP-BIP, and LF-LB for LAMP (details are mentioned in Electronic Supplementary Material S2). For primary proof, N-gene cDNA template was used as a test sample. The cDNA sequence was 466 bp, of which 200 bp of N-gene were specifically targeted during LAMP amplification.

Validation of our system was done with 82 RNA samples derived from clinical swabs, collected at the Robert Koch Institute (RKI in Berlin, Germany). These RNA samples were tested positive for SARS-CoV-2 via real-time qRT-PCR and were in the CT-value range 22 (5.6 × 10^6^ RNA copies/ml) to 33 (3.9 × 10^3^ RNA copies/ml) [20]. The LFA result was analyzed qualitatively by visual readout as positive or negative and semiquantitatively by using a smartphone-based in vitro diagnostic device, which quantified the relative intensities of the test line and control line on the LFA test strip.

## Material and methods

### cDNA-viral RNA template and primer sequences

The specific primers for N-gene PCR amplification and LAMP amplification were designed as mentioned in Electronic Supplementary Material S2 and purchased from Eurofins, Germany. A 466-bp cDNA template was produced by RT-PCR amplification from viral RNA, by a specific set of forward and reverse primers for N-gene. There were 6 primers used in LAMP, namely, F3-B3 (forward–backward outer primer), FIP-BIP (forward–backward inner primer), and LF-LB (loop forming forward–backward primer). The LF primer was FITC-labeled for aiding in detection using the lateral flow assay. The amplification was confirmed by gel electrophoresis and eluted out using PureLink Quick Gel Extraction kit (Invitrogen). Clinical RNA samples from dry nose and throat swab samples were dissolved in PBS, and the solution was thereafter subjected to extraction using QIAamp Viral RNA Mini Kit (Qiagen, Hilden, Germany) following manufacturer’s protocol. RNA was finally eluted out in molecular-grade water and then used as a sample in this study. The viral load of RNA was measured via real-time qRT-PCR targeting E-gene and ORF1ab gene regions, with reference to INSTAND standard by RKI, Berlin, Germany [20].

### LAMP reaction mixture

The reaction mixtures were prepared for 25 μL reaction volume. The cDNA LAMP reaction mixture was prepared as described in Electronic Supplementary Material Table S2, and the RNA LAMP reaction mixture was prepared as described in Electronic Supplementary Material Table S4. The three enzymes *Bst* polymerase 3.0, helicase, and reverse transcriptase were used together to increase the robustness of the assay. Special preparatory precautions were taken and the preparation of the reaction mixture was done on ice (~ 4 °C).

### LAMP program

The LAMP programs for cDNA (see Electronic Supplementary Material Table S3) and RNA (see Electronic Supplementary Material Table S5) were ran in TProfessionalTRIO thermocycler, Biometra.

### Lateral flow assay

Five microliters of the LAMP product was applied to the sample loading area of the test strip with 20 μL of the assay buffer (HybriDetect kit by Milenia Biotech, Germany). The test strip was placed upright so that the conjugate of LAMP product and GNP-anti-FITC-Ab migrated through the strip via capillary flow. The control and test lines on the test strip were read after 2–3 min incubation at room temperature.

### Gel electrophoresis

The LAMP product was run on 2% agarose gels for confirmation of a “ladder like” banding pattern of the LAMP product in 1 × TAE buffer (Invitrogen) at 95 V for ~ 25 min. The gel electrophoresis was visualized with a UV-Visualizer, E-box (Vilber, France).

### Imaging

The images for LFA were captured by iPhone cameras. Semiquantitative analysis of the LFA was performed by a smartphone-based in vitro diagnostic device produced for use in our work by MicroDiscovery GmbH (Berlin, Germany).

## Results and discussion

### Proof of working of LAMP

#### Fluorescent LAMP in a tube

**Fig. 2:**
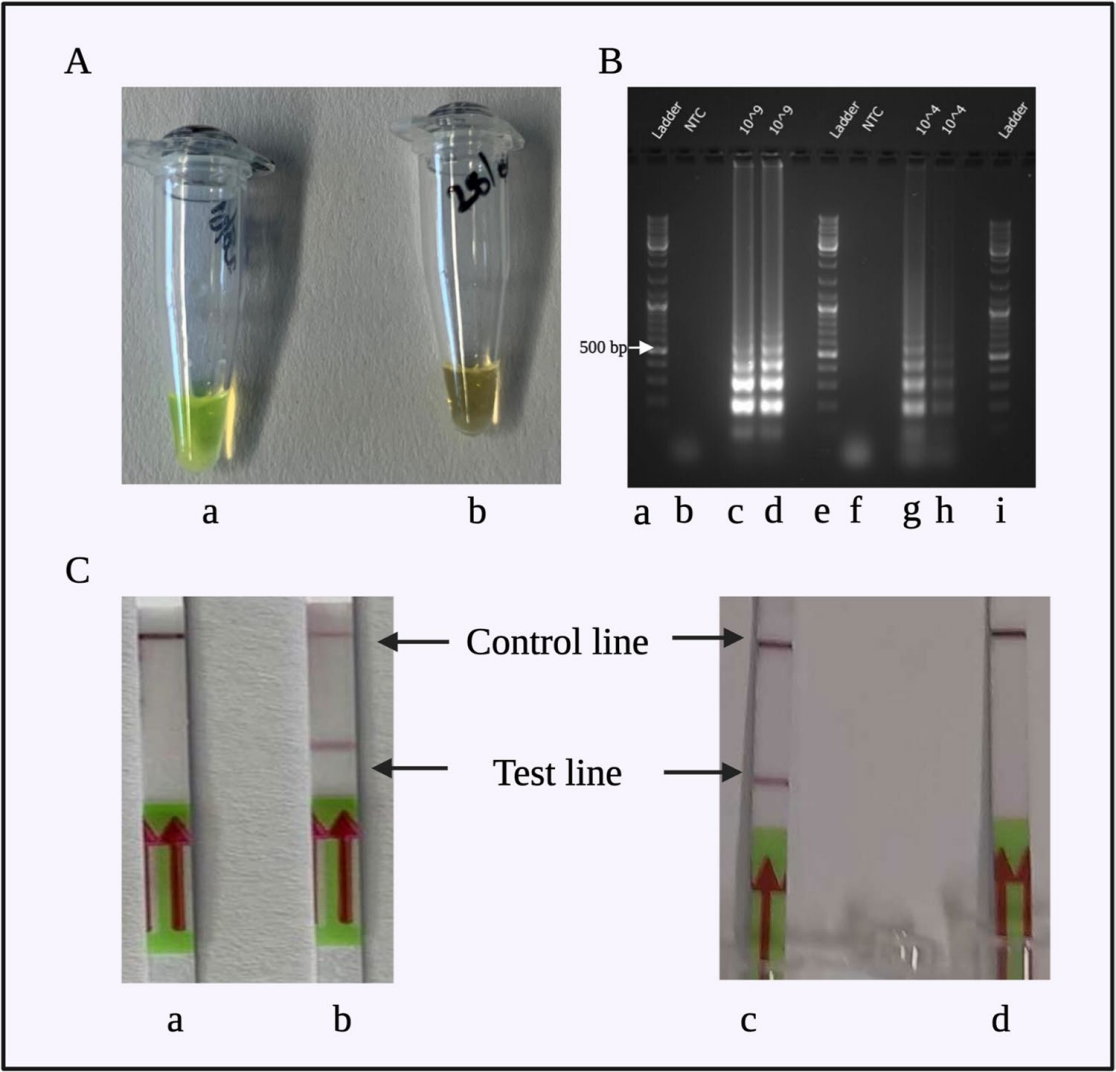
The results of preliminary tests for confirmation of LAMP and LFA compatibility. A Colorimetric LAMP with SYBR-Green produced a bright green color in (a) the positive sample and dull orange color in (b) the non-template control. B LAMP products were observed to show a “ladder like” band pattern when the amplification was successful. Lanes: (a) marker (100 bp), (b) non-template control 1, (c) cDNA LAMP-amplified product with 10^9^ copies/μL, (d) repetitive sample for LAMP-amplified cDNA with 10^9^ copies/μL, (e) marker (100 bp), (f) non-template control 2, (g) cDNA LAMP-amplified product with 10^4^ copies/μL, (h) repetitive sample for LAMP-amplified cDNA with 10^4^ copies/μL, and (i) marker (100 bp). In C, the LAMP product was read out on the test strip. Test and control lines appeared as expected—positive readout: both lines, negative readout: only control line. Stripes/samples: (a) negative readout for the non-template control, (b) positive readout for cDNA with biotin-LB primer and FITC-LF primer, (c) positive readout for cDNA with biotin-dUTP (5%) and FITC-LF primer, and (d) negative readout for the non-template control

LAMP was performed with a primary LAMP protocol for N-gene cDNA detection. Twenty-five microliters of LAMP reaction volume constituted of the ingredients mentioned in Electronic Supplementary Material Table S6. We used different primer concentrations: F3 and B3 (0.6 μM), LF and LB (0.4 μM), and FIP and BIP (1.6 μM). *Bst* 3.0 polymerase was added to the reaction mixture at a final concentration of 0.32 U/μL. LAMP program was a single cycle of 40 min (65 °C for 30 min, 80 °C for 5 min, and 4 °C for at least 5 min). After LAMP amplification, SYBR-Green was added to the product to look for fluorescence or colorimetric signals. A bright green-yellow coloration was observed for the DNA sample and a dull orange coloration for the non-template control (NTC). The result was also visible with the naked eye (Fig. 2A).

### LAMP readout via LFA

LAMP was modified to be read on the lateral flow test strips. Initially, FITC-labeled LF primer and biotin-labeled LB primer were used. LAMP was performed as described in Electronic Supplementary Material S3. LFA was performed as described in “Conclusion and summary.” The test strip readout indicated a clear positive result for the cDNA sample by producing two lines on the LFA test strip, while the NTC produced only one line on the LFA test strip (Fig. 2C(a, b)). This confirmed the compatibility of LAMP and LFA. However, replication experiments showed that the reproducibility was low due to unspecific amplification. As also reported previously, NTC is known to show unspecific amplification [9], so modifications in the LAMP reaction were made.

#### *Bst* 2.0 polymerase vs *Bst* 3.0 polymerase

WarmStart (with *Bst* 2.0 polymerase) RT-LAMP master mix (New England, Biolabs) and *Bst* 3.0 polymerase were tested in parallel to check the difference in the two enzyme activities. We observed that the master mix was less robust and produced more unspecific amplification as compared to *Bst* 3.0 polymerase. The combined efficacy and robustness of *Bst* 3.0 polymerase was the reason why it was chosen for all further experiments. The reaction mixture and protocol were modified accordingly.

### LAMP modifications

#### Incorporating biotin-11-dUTPs to increase specificity of LAMP

For a reproducible LAMP-LFA readout, it is necessary to efficiently incorporate FITC and biotin labels into the amplification products. Firstly, we tested a combination of FITC-dUTP with biotin-LB primer but observed that FITC-dUTP is not incorporated during amplification using *Bst* 3.0 polymerase, for a 10-min LAMP annealing time. This likely is due to the FITC label hampering the activity of *Bst* 3.0 or FITC-labeled dUTP takes a longer time to incorporate. Consequently, we tested a combination of biotin-dUTP and FITC-LF primer. Biotin-dUTP was investigated for 1%, 5%, 10%, and 20% of dNTP volume in the 25 μL reaction, for 10-min LAMP annealing time. We assessed that 5% B-dUTPs gave us the best test band on the LFA (Fig. 2C(c, d)). The NTC gave a clear negative control, and hence, the reaction mixture was established for further experiments. In the abovementioned work by Tan et al., the incorporation of FITC-labeled dUTP during LAMP amplification in 30 min was reported [19]; however, we were not able to reproduce these results with *Bst* 3.0 polymerase in 10 min.

#### LFA compatibility

The LFA test strips used are standardized and stabilized for use at room temperature. For the LAMP product readout compatibility with LFA, the LF primer was tagged with the FITC label, generating a FITC-flanked region in the amplified LAMP product. Biotin-dUTPs were incorporated during elongation and generated biotin flanking regions in the amplified LAMP product. FITC in the amplified product was captured by GNP-anti-FITC-Ab conjugate at the sample loading area on the test strip. The agglomeration of GNPs on the test band and control band aided in visual readout on the LFA (Fig. 2C(c, d)). This result was obtained reproducibly and confirmed with the gel electrophoresis results (Fig. 2B).

#### Enzyme mixture for increasing robustness and specificity of LAMP

Since *Bst* 3.0 already has reverse transcriptase activity, RT-LAMP may be performed with only a single enzyme. Although the enzyme is self-sufficient, we added reverse transcriptase (RTase, 1 μL) to enhance reverse transcriptase activity, to increase the robustness of RNA amplification via RT-LAMP. Also, the non-specificity of LAMP needed to be reduced. It was noted that the primers in LAMP possibly dimerize and amplify, therefore generating unspecific NTC or background signals. The generation of false positives was prevented by using helicase (1 μL) in the LAMP enzyme mixture. Helicase is an ATP-dependent enzyme; hence, 1 μL of ATP (10 mM) was added to the LAMP reaction mixture. The addition of RTase decreases RNA amplification by 5 min, which brought the overall RT-LAMP reaction time to 15 min. Besides reducing the amplification time, the enzyme mixture did not cause any adverse effect on the readout of LFA.

### Establishing LAMP-LFA with viral cDNA

#### Standardization of the LAMP protocol

To establish the LAMP-LFA detection method, N-gene cDNA was used as template for LAMP amplification. The target in LAMP was a 200-bp section out of the 466-bp N-gene. LAMP was performed as described in “LAMP reaction mixture” and “LAMP program.” LAMP performance was analyzed at various amplification annealing time intervals, starting with 5 min up to 25 min (in steps of 5 min). For each sample, a LFA was tested along with confirmation of amplification with 2% agarose-gel electrophoresis. Different primer concentrations for each set of primers were also analyzed and then fixed for all future experiments, as described in Electronic Supplementary Material S3. The report by Tan et al. reports a working LAMP using a reduced 4 primer set (F3-B3 and FIP-BIP) [19]. We also tried to use a primer-reduced set, but the system does not perform reliably with our LAMP system. The 6-primer system worked specifically for N-gene target in our work (Electronic Supplementary Material S2).

#### LFA readout for cDNA LAMP

**Fig. 3:**
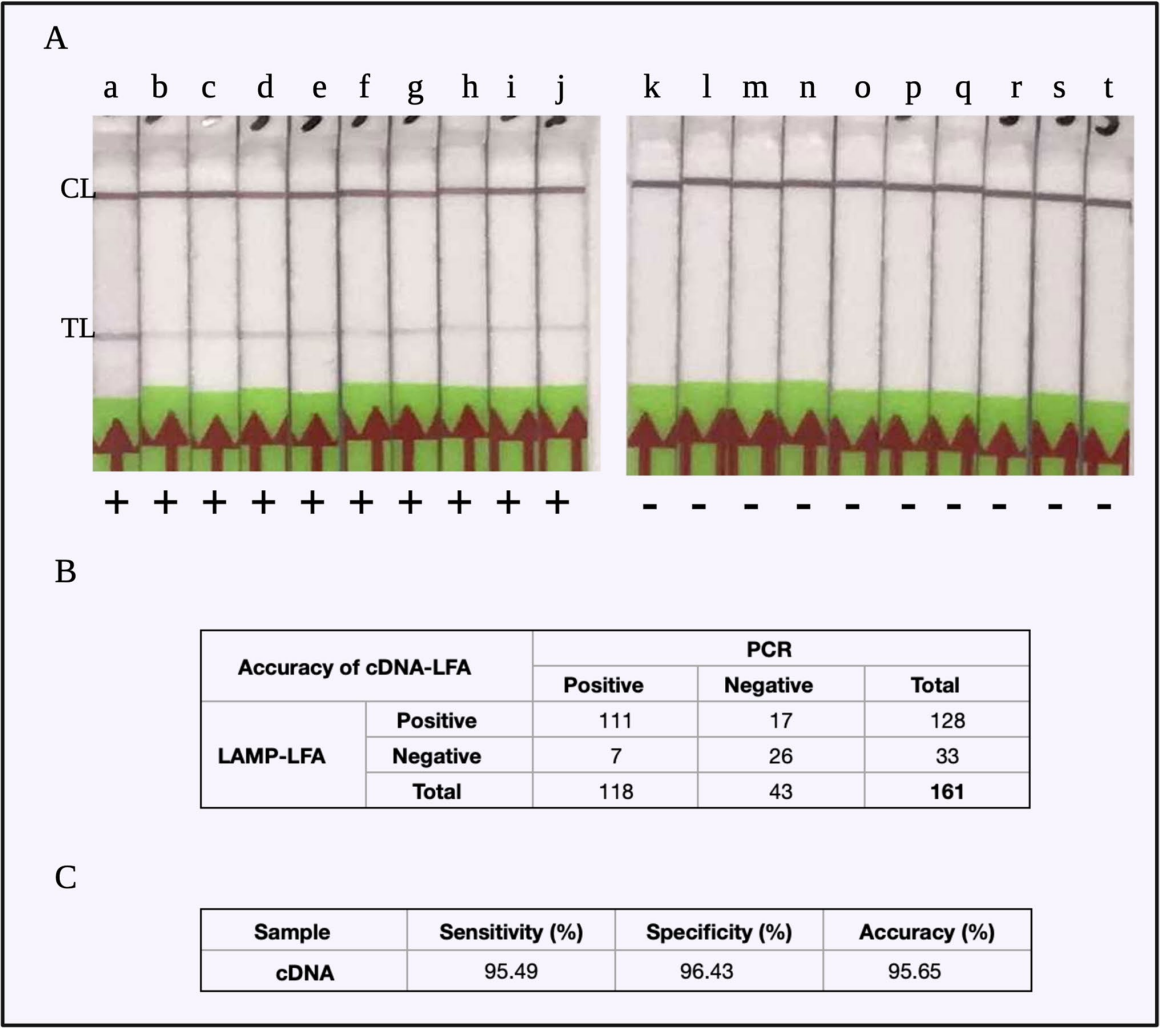
LAMP-LFA experiments with cDNA. A LFA readout for (a) to (j): cDNA LAMP-amplified product, showing a clear control line (CL) and test line (TL); (k) to (t): non-template controls for each of the cDNA LAMP products, showing only control lines (CL). B The contingency table for all the cDNA LAMP experiments, where a total of 161 samples were tested. C The sensitivity = 95.49%, specificity = 96.43%, and accuracy = 95.65% of the cDNA-LAMP-LFA results

cDNA detection was analyzed with two distinct tests and control lines on the LFA test strip. The test line is formed only when there is amplification of the template cDNA, and the control line is always seen as the reference to the validity of test strips. Non-template controls (NTCs) were observed to produce only one control line on the test strip, implying no amplification (Fig. 3A). Each experiment was performed with NTC for verifying the specificity of the cDNA reactions. A LAMP amplification time of 10 min was sufficient for an unambiguous LFA result for cDNA as template and was therefore fixed for consecutive experiments.

#### Statistical inference of cDNA-LAMP-LFA

To determine reproducibility of the assay, LAMP experiments were carried out for a total of 161 samples, out of which 28 were NTC and 133 were cDNA. The assay was 95.49% sensitive (Clopper-Pearson 95% CI of 90.44 to 98.33%) and 96.43% specific (with Clopper-Pearson 95% CI of 81.65 to 99.91%) (Fig. 3B). By using the McNemar statistical test for a paired nominal dataset, we confirmed statistically that the sensitivity of LAMP-LFA results corresponds significantly to the specificity of the assay (*p*-value = 0.064). Therefore, we determined that our LAMP protocol could be applied for viral RNA detection.

### Proof of viral RNA detection via RT-LAMP-LFA

#### Target specificity confirmation

Eighty clinical RNA samples from patients that tested positive for SARS-CoV-2 were obtained with different CT values (confirmed by real-time qRT-PCR). The extracted RNA was obtained directly from human swab samples, so it contained a mixture of viral and human RNA. A human RNA control from 293 T HEK cells was tested with the LAMP primers to confirm no unspecific amplification of human RNA. The human RNA from 293 T HEK cells was used as negative controls. The RNA samples (obtained from RKI) were eluted in molecular-grade water for preservation and further experimental use. The elution water and some other elution buffers were tested as negative controls too.

#### Statistical inference of RT-LAMP-LFA

**Fig. 4:**
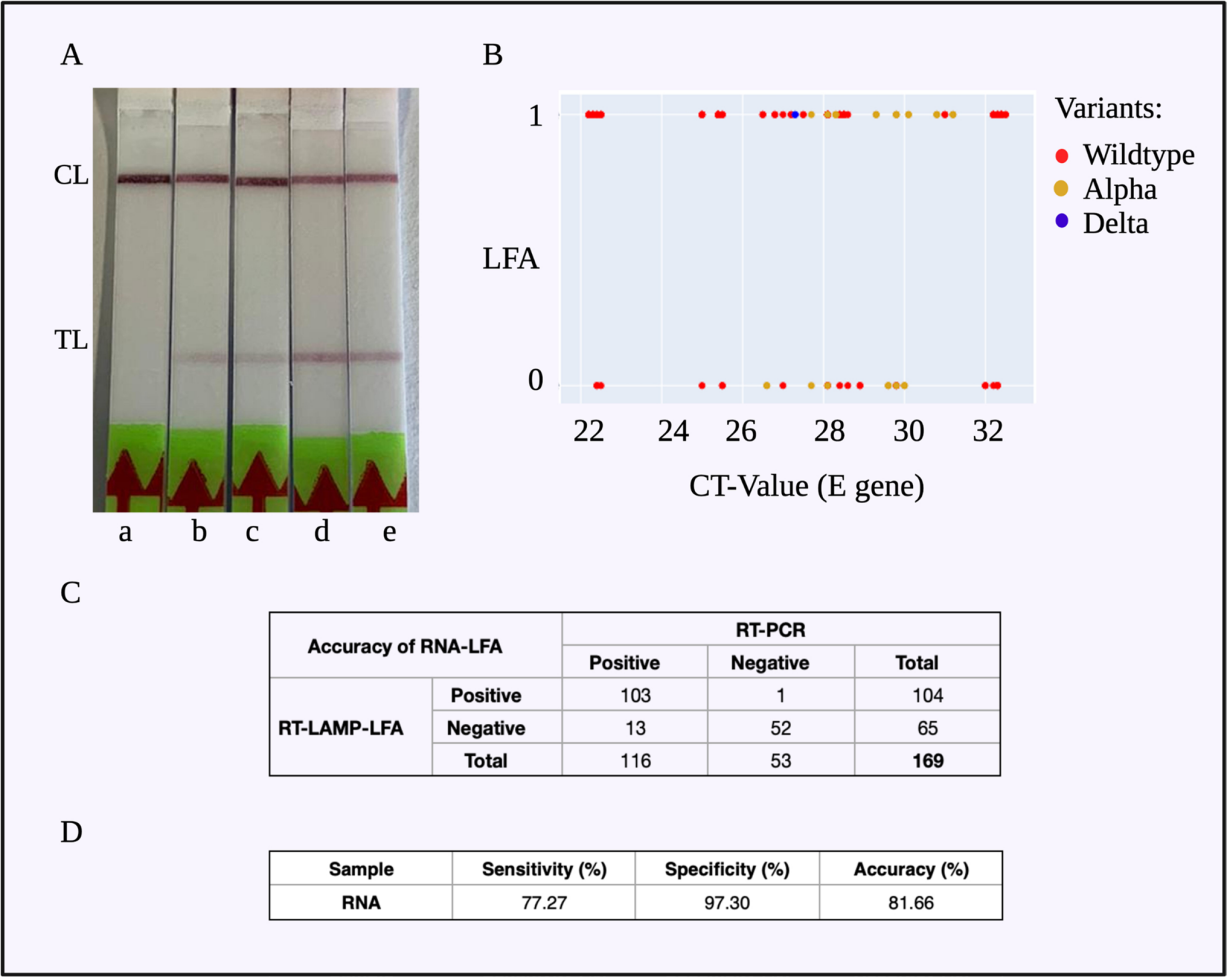
RT-LAMP-LFA experiments with RNA extracted from clinical COVID-19-positive swab samples. A The RT-LAMP-LFA readout for (a) non-template control, (b) RNA with CT-33, (c) RNA with CT-29, (d) RNA with CT-26, (e) RNA with CT-22. The test lines are visible for the RNA-positive samples, while the NTC only showed the control line. Out of the SARS-CoV-2 variants tested with RT-LAMP-LFA, B the variants of concern alpha and delta were detected by our assay system similar to the wild-type strain. C The contingency table for the 169 RT-LAMP-LFA experiments. D The sensitivity = 77.27%, specificity = 97.30%, and accuracy = 81.66% of the clinical RNA RT-LAMP-LFA results

A wide range of CT values was tested, corresponding to 5.6 × 10^6^ RNA copies/ml (CT 22) and 3.9 × 10^3^ RNA copies/ml (CT 33) (Fig. 4A). RT-LAMP was performed for each sample in duplicates and for some in triplicates. It was observed that RNA was detected with RT-LAMP-LFA for all the RNA CT values tested, with 77.27% sensitivity (Clopper-Pearson 95% CI of 69.17 to 84.11%) and 97.30% specificity (Clopper-Pearson 95% CI of 85.84 to 99.93%) (Fig. 4C).

RT-LAMP-LFA was performed in a triplicate for CT-33 (3.9 × 10^3^ RNA copies/ml) and the system could detect the CT-33 RNA with 100% sensitivity (Clopper-Pearson 95% confidence interval of 29.24 to 100.00%) and 100% specificity (Clopper-Pearson 95% confidence interval of 2.50 to 100.00%).

#### Variants of concern investigated

The RNA samples covered different variants of SARS-CoV-2, mainly wild type and VoCs alpha and delta. RT-LAMP could amplify and LFA could detect each of these variants, irrespective of the mutations, using the N-gene specific LAMP primers (Fig. 4B). The efficiency of the RT-LAMP-LFA was comparable with that of real-time qRT-PCR but will be improved with further experimentation.

### Semiquantitative digital analysis of the LFA test strip

The quantification of LFA test and control lines was performed by using a smartphone-based in vitro diagnostics (IVD) device. The device has two main parts: (a) smartphone connected to a (b) test strip holder. The test strip is placed in the test strip slot and the result is visualized and saved on the smartphone. The results of the IVD device are presented in Table 1. Each row presents the analysis of one LFA. The readout from the device is relative intensity values. The relativity measure is to the test strip non-colored space next to the test and control lines.

**Table 1:** The LFA results were read via a smartphone-based in vitro diagnostics device. The device reads relative intensities of the control line and test line produced on the LFA. (A) Intensity readouts from the device for NTC and cDNA LFA results. The intensity of the test line decreases as the concentration of the sample decreases. The ratio of test line vs control line is a measure for the difference of intensities between the test and control lines for each LFA. (B) Relative intensity measures of control lines and test lines for NTC and RNA LFA results

Sample	Sample conc	Test line intensity	Control line intensity	Test/control ratio
A				
NTC	n.a	0.08	59.21	0.00
		0.00	53.44	0.00
		0.00	64.45	0.00
cDNA	10^8^ copies/µL	11.61	26.49	0.44
	10^6^ copies/µL	7.61	29.48	0.26
	10^4^ copies/µL	8.32	46.08	0.18
	10^2^ copies/µL	0.49	56.78	0.01
B				
NTC	n.a	0.00	58.66	0.00
		0.19	62.61	0.00
		0.00	43.53	0.00
RNA	CT-27	11.44	43.29	0.26
	CT-27	11.73	46.51	0.25
	CT-30	10.79	50.55	0.21
	CT-30	6.34	57.16	0.11

## Conclusion and summary

In this communication, we present an RT-LAMP-LFA technology for the sensitive, reproducible detection of SARS-CoV-2 cDNA and RNA based on the viral N-gene. The LOD for viral RNA was found to be 3.9 × 10^3^ RNA copies/ml with the amplification as well as the assay development time being as short as 15 min. Additionally, the smartphone-based readout of the LFA bridges the gap for a digital confirmation of the visual readout and enables a semiquantitative analysis of the results. The potential of LAMP technology for POCT is currently impeded by the use of *Bst* 3.0 polymerase operating at temperatures higher than 60 °C. Thus, we will develop our method further with respect to using alternative polymerases (e.g., *Taq*) which can work efficiently at more ambient temperatures. Moreover, swab samples will be tested to demonstrate the applicability of this assay with real samples.

Combining both, LAMP and LFA, in one assay creates a versatile technology with options for the sensitive POC detection of various other infectious diseases (viral, bacterial, fungal, or parasitic) and rare diseases and perhaps for any nucleic acid as biomarker.

## Supplementary Information

Below is the link to the electronic supplementary material.

Supplementary file1 (PDF 176 KB)
