# Supplementary material for: Lateral flow–based nucleic acid detection of SARS-CoV-2 using enzymatic incorporation of biotin-labeled dUTP for POCT use
Source: Anal Bioanal Chem. 2022 Jan 19;414(10):3177–86. doi: 10.1007/s00216-022-03880-4 (PMC8766626; doi:10.1007/s00216-022-03880-4)
Supplement: Supplementary file 1 — Supplementary file1 (PDF 176 KB) [file 216_2022_3880_MOESM1_ESM.pdf]

Lateral flow–based nucleic acid detection of SARS-CoV-2 using enzymatic incorporation of biotin- labeled dUTP for POCT use

Journal: Analytical and Bioanalytical Chemistry

Saloni Agarwal[1], Christian Warmt[2], Joerg Henkel[2], Livia Schrick[3], Andreas Nitsche[3], Frank F. Bier[1,4]

1. University of Potsdam, Institute for Biochemistry and Biology, Chair of Molecular Bioanalysis and Bioelectronics, Karl-Liebknecht-Strasse 24/25, 14476 Potsdam (Golm), Germany
Email: fbier@uni-potsdam.de, agarwal@uni-potsdam.de

2. Fraunhofer-Institute for Cell Therapies and Immunology, Branch Bioanalysis and Bioprocesses, IZI-BB,
Am Mühlenberg 13, 14476 Potsdam (Golm), Germany
E-mail: christian.Warmt@izi-bb.fraunhofer.de, joerg.henkel@izi-bb.fraunhofer.de

3. Robert Koch Insitute, Centre for Biological Hazards and Special Pathogens,
Seestr. 10, 13353 Berlin, Germany
E-mail: SchrickL@rki.de, NitscheA@rki.de

4. Institute for Molecular Diagnostics and Bioanalysis – IMDB gGmbH,
Veltener Str. 12, 16761 Hennigsdorf b. Berlin, Germany

Corresponding author: Prof. Frank Bier (fbier@uni-potsdam.de)

Supplementary Material:

S1: DNA concentrations used in experiments

S2: LAMP and PCR primers

S3: cDNA LAMP details

S4: RNA RT-LAMP details

S5: Methodology for colorimetric LAMP

S1: DNA concentrations used in the experiments-

1) 13.5 ng/µL (2.7 x 10^{10} copies/µL)
2) 15.7 ng/µL (3.1 x 10^{10} copies/µL)
3) 41.5 ng/µL (8.2 x 10^{10} copies/µL)
4) 39.5 ng/µL (7.9 x 10^{10} copies/µL)
5) 41.3 ng/µL (8.2 x 10^{10} copies/µL)
6) 30.6 ng/µL (6.1 x 10^{10} copies/µL)
7) 13.2 ng/µL (2.6 x 10^{10} copies/µL)

S2: LAMP and PCR Primers-

cDNA was prepared from viral N-gene. The following sequence is of 466 bp length and the primers have been highlighted in the sequence with corresponding colours in the Table S1. These were prepared using PrimerExplorer.

5'GACCCCAAAATCAGCGAAATGCACCCCGCATTACGTTTGGTGGACCCTCAGATTCAAC
TGGCAGTAACCAGAATGGAGAACGCAGTGGGGCGCGATCAAAACAACGTCGGCCCCAA
GGTTTACCCAATAATACTGCGTCTTGGTTCACCGCTCTCACTCAACATGGCAAGGAAGAC
CTTAAATTCCCTCGAGGACAAGGCGTTCCAATTAACACCAATAGCAGTCCAGATGACCA
AATTGGCTACTACCGAAGAGCTACCAGACGAATTCGTGGTGGTGACGGTAAAATGAAAG
ATCTCAGTCCAAGATGGTATTTCTACTACCTAGGAACTGGGCCAGAAGCTGGACTTCCCT
ATGGTGCTAACAAAGACGGCATCATATGGGTTGCAACTGAGGGAGCCTTGAATACACCA
AAAGATCACATTGGCACCCGCAATCCTGCTAACAATGCTGCAATCGTGCTACA-3'

Primers	Sequences (5' -> 3')	Length (nt)
F3	CCCAAAATCAGCGAAATGCA	20
B3	AGCCAATTTGGTCATCTGGA	20
FIP	TGTTTTGATCGCGCCCCACTGATTACGTTTGGTGGACCCTC	41
BIP	TGCGTCTTGGTTCACCGCTCATTGGAACGCCTTGTCCTC	39
**FITC**-LF	**FITC—**TCCATTCTGGTTACTGCCAGTTGAA	25
LB	ACTCAACATGGCAAGGAAGACCTTA	25
Fwd_PCR	GACCCCAAAATCAGCGAAAT	20
Rev_PCR	TGTAGCACGATTGCAGCATTG	20

Table S1: Sequence of Primers for RT-LAMP N-gene.

S3: cDNA LAMP details-

S3.1: LAMP reaction mixture for DNA samples (Table S2)-

a) All the ingredients are vortexed and centrifuged for each 2-3 seconds.
b) Enzymes and DNA are brought to the working place after the master mix is prepared.
c) The ingredients are mentioned in the sequence of addition in the reaction mix.
d) PCR oil was layered on top of the whole reaction mixture to prevent unwanted contamination.
e) All reaction mixtures are prepared on ice.

**cDNA-LAMP 25 µL Reaction mixture**		
	**Ingredients (Std. Conc)**	**Volume (end Conc.)**
Master Mix	Isothermal buffer (10X)	2.5 µL (1X)
	$MgSO_4$ (100mM)	1.5 µL (6mM)
	dNTPs (40mM)	2.9 µL (5mM)
	Biotin-dUTP (1 mM)	3.1 µL (5%, 1:20 dilut.)
	F3 Primer (10µM)	1.5 µL (0.6 µM)
	B3 Primer (10µM)	2.5 µL (1µM)
	FIP Primer (10 µM)	1.5 µL (0.6 µM)
	BIP Primer (10 µM)	1.5 µL (0.6 µM)
	FITC-LF Primer (10 µM)	2.5 µL (1µM)
	LB Primer (10 µM)	1.5 µL (0.6 µM)
Enzymes	ATP (10mM)	1 µL (0.4 µM)
	Helicase (200nM)	1 µL (4mM)
	Bst 3.0 Polymerase (8U/µL)	1 µL (0.32 U/µL)
Sample	cDNA	1 µL
	Nuclease free water	Volume up to 25 µL

Table S2: cDNA LAMP reaction mixture ingredients concentration and volume chart

S3.2: LAMP Program for cDNA samples (Table S3)-

**LAMP Program**	
**Step**	**Temp (Time)**
Annealing and extension	65°C (10 min)
Inactivation	85°C (3 min)
Cooling	4°C (storage)

Table S3: cDNA LAMP reaction temperature and time chart

S4: RNA RT-LAMP details-

S4.1: RT-LAMP mixture for RNA samples (Table S4)-

a) All the ingredients are vortexed and centrifuged for each 2-3 seconds.
b) Enzymes and DNA are brought to the working place after the master mix is prepared.
c) The ingredients are mentioned in the sequence of addition in the reaction mix.
d) PCR oil was layered on top of the whole reaction mixture to prevent unwanted contamination.
e) All reaction mixtures are prepared on ice.

RNA-LAMP 25 µL Reaction mixture		
	**Ingredients (Std. Conc)**	**Volume (end Conc.)**
Master Mix	Isothermal buffer (10X)	2.5 µL (1X)
	$MgSO_4$ (100mM)	1.5 µL (6mM)
	dNTPs (40mM)	2.9 µL (5mM)
	Biotin-dUTP (1 mM)	3.1 µL (5%, 1:20 dilut.)
	F3 Primer (10µM)	1.5 µL (0.6 µM)
	B3 Primer (10µM)	2.5 µL (1µM)
	FIP Primer (10 µM)	1.5 µL (0.6 µM)
	BIP Primer (10 µM)	1.5 µL (0.6 µM)
	FITC-LF Primer (10 µM)	2.5 µL (1µM)
	LB Primer (10 µM)	1.5 µL (0.6 µM)
Enzymes	ATP (10mM)	1 µL (0.4mM)
	Helicase (200nM)	1 µL (4mM)
	RTase (15 U/µL)	1 µL (0.6 U/µL)
	Bst 3.0 Polymerase (8U/µL)	1 µL (0.32 U/µL)
Sample	RNA	1µL
	Nuclease free water	Volume up to 25 µL

Table S4: RNA LAMP reaction mixture ingredients concentration and volume chart

S4.2: RT-LAMP Program for RNA Samples (Table S5)-

LAMP Program	
**Step**	**Temp (Time)**
Annealing and extension	65°C (15 min)
Inactivation	85°C (3 min)
Cooling	4°C (storage)

Table S5: RNA RT-LAMP reaction temperature and time chart

S5: Methodology for colorimetric LAMP:

1. Allow the DNA to thaw on ice in a separate ice container (this ice container must not be near the LAMP workstation). The smallest contamination leads to false positive results
2. Put on new gloves
3. Always work on ice
4. Label PCR eppendorfs for the different approaches and put them on ice so that they can are pre-cooled
5. Pipette the master mix together in the following order: (**Table S6**)
   a. Nuclease-free H2O
   b. Isothermal buffer
   c. MgSO4
   d. DMSO
   e. dNTP mix
   f. F3 primer (please use this for small numbers of samples due to the small volume pipette up and down 5x when draining)
   g. B3 primer (please use here for small numbers of samples due to the small volume pipette up and down 5x when draining)
   h. FIP primer
   i. GDP primer
   j. FL primer (here due to the small volume for small numbers of samples, please drain up and down 5x)
   k. BL-Primer (here due to the small volume for small numbers of samples please pipette up and down 5x when draining)
6. Re-freeze all master mix components except the water
7. Bring the enzyme to the workplace at the same time
8. Vortex enzyme briefly (time: 2 sec) at low level (level: 3)
9. Draw up the required volume of enzyme with the pipette (leave the tip briefly in the vessel until the Enzyme solution has been completely absorbed)
10. Pipette enzyme into the master mix (1st pressure point) and pipette 2-3 up and down, then drain the entire volume (2nd pressure point)
11. Remove enzyme
12. Vortex the master mix for 3 sec (level: 8) and centrifuge briefly (time: 3 sec)
13. Distribute the master mix of 23.5 µl per sample to the prepared eppis
14. Pipette 1.5 µl nuclease-free H2O into the NTC and 50 µl PCR oil onto the sample. Pipette on it, close the lid of the eppis and place on ice (Oil is used to prevent contamination of the room when the vessels are opened later is minimised or prevented)
15. Take away the water
16. Only now is the DNA brought to the workplace! Vortex DNA (level: 8; time: 4 sec) and centrifuge briefly (time: 3 sec)
17. Pipette 1.5 µl of DNA into the samples provided, pipette up and down 10 times
18. Seal samples with 50 µl PCR oil
19. Place samples in the cycler (TRobot from Analytik Jena) and start LAMP
20. LAMP program: (lid heating to 90°C)
21. Let the samples cool for at least 10 minutes at 4°C in the cycler
22. Take samples from the cycler and perform SYBR-Green detection
23. For the SYBR-Green detection, 1 µl of 1000X SYBR-Green is passed through the oil layer into the LAMP reaction mixture pipette.

24. To mix, pipette up and down 10 times with the volume set on the pipette (without oil; pipette through the oil) and, if necessary, snap on the eppi 2-3 times
25. Evaluate under UV lamp (UVis, Desaga) at 366 nm

**Colorimetric-LAMP 25 µL reaction mixture**		
	**Ingredients (Std. Conc)**	**Volume (end Conc.)**
Master Mix	Isothermal buffer (10X)	2.5 µL (1X)
	$MgSO_4$ (100mM)	1.5 µL (6mM)
	dNTPs (40mM)	3.5 µL (5.6 mM)
	F3 Primer (10µM)	0.5 µL (0.2 µM)
	B3 Primer (10µM)	0.5 µL (0.2 µM)
	FIP Primer (10 µM)	4 µL (1.6 µM)
	BIP Primer (10 µM)	4 µL (1.6 µM)
	LF Primer (10 µM)	1 µL (0.4 µM)
	LB Primer (10 µM)	1 µL (0.4 µM)
	DMSO (99.8%)	1.9 µL (7.9%)
Enzymes	*Bst* 3.0 Polymerase (8U/µL)	1 µL (0.32 U/µL)
Dye	SYBR-Green (50X)	1 µL (2X)
Sample	DNA	1.5 µL
	Nuclease free water	Volume up to 25 µL

Table S6: LAMP reaction mixture ingredients concentration and volume chart for colorimetric LAMP assay in a tube.
